# EUROCRINE®: Nebennierenoperationen 2015 bis 2019 – überraschende erste Ergebnisse

**DOI:** 10.1007/s00104-020-01277-6

**Published:** 2020-09-18

**Authors:** J. I. Staubitz, T. Clerici, P. Riss, F. Watzka, A. Bergenfelz, E. Bareck, V. Fendrich, A. Goldmann, F. Grafen, A. Heintz, R. M. Kaderli, E. Karakas, B. Kern, M. Matter, M. Mogl, C. A. Nebiker, B. Niederle, J. Obermeier, A. Ringger, R. Schmid, F. Triponez, A. Trupka, C. Wicke, T. J. Musholt

**Affiliations:** 1 Sektion Endokrine Chirurgie der Klinik für Allgemein-, Viszeral- und Transplantationschirurgie, Universitätsmedizin Mainz, Langenbeckstraße 1, Mainz, 55131 Deutschland; 2 Kantonsspital St. Gallen, St. Gallen, Schweiz; 3 Universitätsklinik für Chirurgie, Medizinische Universität Wien, Wien, Österreich; 4 Chirurgie, Lund University, Lund, Schweden; 5 Abteilung für Chirurgie, KRAGES Burgenländische Krankenanstalten-Ges.m.b.H., Oberpullendorf, Österreich; 6 Klinik für Endokrine Chirurgie, Schön Klinik Hamburg Eilbek, Hamburg, Deutschland; 7 Viszeral- und Thoraxchirurgie, Kantonsspital Winterthur, Winterthur, Schweiz; 8 Chirurgische Klinik, Spital Limmattal, Schlieren, Schweiz; 9 Allgemein- und Viszeralchirurgie, Katholisches Klinikum Mainz, Mainz, Deutschland; 10 Viszerale Chirurgie, Universitätsspital Bern, Bern, Schweiz; 11 Klinik für Allgemein‑, Visceral- und Endokrine Chirurgie, Krankenhaus Maria Hilf Krefeld, Krefeld, Deutschland; 12 Viszeralchirurgie, St. Claraspital Basel, Basel, Schweiz; 13 Chirurgie Viscérale, Centre Hospitalier Universitaire Vaudois, Lausanne, Schweiz; 14 Chirurgische Klinik, Charité Universitätsmedizin Berlin, Berlin, Deutschland; 15 Viszeralchirurgie, Kantonsspital Aarau, Aarau, Schweiz; 16 Abteilung für Chirurgie, Franziskus Spital Wien, Wien, Österreich; 17 Klinik für Chirurgie, Klinikum Dortmund gGmbH, Dortmund, Deutschland; 18 Chirurgie, Solothurner Spitäler AG, Solothurn, Schweiz; 19 Viszeralchirurgie, Spitalzentrum Biel, Biel, Schweiz; 20 Chirurgie thoracique et endocrinienne, Hôpitaux Universitaires Genève, Genève, Schweiz; 21 Chirurgische Klinik, Klinikum Starnberg, Starnberg, Deutschland; 22 Kantonsspital Luzern, Luzern, Schweiz

**Keywords:** Adrenalektomie, Laparoskopische Adrenalektomie, Retroperitoneoskopische Adrenalektomie, EUROCRINE®-Register, Nebennierenmetastase, Nebennierenrindenkarzinom, Adrenalectomy, Laparoscopic adrenalectomy, Retroperitoneoscopic adrenalectomy, EUROCRINE® registry, Adrenal metastasis, Adrenocortical carcinoma

## Abstract

**Hintergrund:**

Seit 2015 erfolgt in Europa mithilfe des EUROCRINE®-Registers eine systematische Dokumentation endokrin-chirurgischer Operationen. Ziel dieser ersten Auswertung war eine Darstellung der Versorgungsrealität für Nebenniereneingriffe in einem homogenen Versorgungsumfeld, entsprechend des deutschsprachigen Raums – bzw. des Präsenzgebiets der Chirurgischen Arbeitsgemeinschaft Endokrinologie (CAEK) der Deutschen Gesellschaft für Allgemein- und Viszeralchirurgie (DGAV) – einschließlich einer Analyse der Adhärenz zu geltenden Therapieempfehlungen.

**Methodik:**

Es erfolgte eine deskriptive Analyse der präoperativen Diagnostik, der angewandten Operationstechniken sowie der zugrunde liegenden histologischen Entitäten der zwischen den Jahren 2015 und 2019 über EUROCRINE® in Deutschland, Österreich und der Schweiz dokumentierten Nebennierenoperationen.

**Ergebnisse:**

In den insgesamt 21 teilnehmenden Kliniken des deutschsprachigen EUROCRINE®-Gebiets wurden 658 Operationen an Nebennieren durchgeführt. In 90 % erfolgten unilaterale, in 3 % bilaterale Adrenalektomien und in 7 % andere Resektionsverfahren. Die in 41 % der Operationen dokumentierte histologische Hauptdiagnose war das adrenokortikale Adenom. In 15 % lagen maligne Befunde zugrunde (einschließlich 6 % Nebennierenrindenkarzinome (ACC) und 8 % Nebennierenmetastasen). 23 % der Operationen erfolgten bei Phäochromozytomen. Diese wurden zu 82 % minimal-invasiv operiert, Nebennierenrindenkarzinome lediglich zu 28 % und Nebennierenmetastasen zu 66 %.

**Schlussfolgerung:**

Überraschenderweise wurden nach Nebennierenadenomen und Phäochromozytomen an dritthäufigster Stelle Nebennierenmetastasen unterschiedlicher Primärtumoren reseziert. 28 % der ACC waren für minimal-invasive Techniken vorgesehen, wobei 20 % dieser Fälle eine Konversion zur offenen Operation erforderten. Die aktuelle Analyse deckte Diskrepanzen zwischen Versorgungsrealität und Leitlinienempfehlungen auf, aus denen sich zahlreiche Fragestellungen ergeben, welche nun in ein überarbeitetes EUROCRINE®-Modul zur Dokumentation von Nebennierenoperationen einfließen werden.

## Abstract (en)

**Background:**

Since 2015 operations performed in the field of endocrine surgery have been entered into the European registry EUROCRINE®. The aim of this analysis was a description of the current healthcare situation for adrenal surgery in a homogeneous healthcare environment corresponding to the German-speaking countries—or to the presence of the working group on surgical endocrinology (CAEK) of the German society for general and visceral surgery (DGAV)—and to assess the adherence to current international treatment guidelines.

**Methods:**

An analysis of the preoperative diagnostics, the applied operative techniques and the underlying histological entities was carried out for all operations on adrenal glands in Germany, Switzerland and Austria, which were registered in EUROCRINE® from 2015 to 2019.

**Results:**

In the total of 21 participating hospitals from the German-speaking EUROCRINE® countries, 658 operations on adrenal glands were performed. In 90% of cases unilateral adrenalectomy was performed, in 3% bilateral adrenalectomy and in 7% other resection procedures. In 41% the main histological diagnosis was an adrenocortical adenoma. In 15% malignant entities were detected on final histology, including 6% adrenocortical carcinoma (ACC) and 8% metastases to the adrenal glands. 23% of the operations were performed for pheochromocytoma. This entity was primarily resected using minimally invasive approaches (82%), whereas minimally invasive techniques were applied in 28% for ACC and in 66% for metastases to the adrenal glands.

**Conclusion:**

Surprisingly, following adrenocortical adenoma and pheochromocytoma, the third most common histological entity was metastasis of different extra-adrenal primary tumors to the adrenal gland. Of the operations for ACC 28% were scheduled for minimally invasive techniques, but conversion to open surgery was necessary in 20%. The analysis revealed discrepancies between treatment reality and international guideline recommendations that raise questions, which will be addressed by an updated version of the EUROCRINE® module for the documentation of adrenal surgery.

## Hintergrund

Das europäische Register EUROCRINE® ist seit dem Jahr 2015 für Kliniken mit endokrin-chirurgischem Schwerpunkt verfügbar. Im Rahmen des „Health Programme“ der Europäischen Union wurde das Projekt im Jahr 2013 initiiert. Seit dem Jahr 2018 ist die EUROCRINE® Society mit dem Sitz in Wien als Non-profit-Organisation registriert. EUROCRINE® steht unter der Leitung eines Steuergremiums, welches sich aus Repräsentanten der nationalen chirurgischen Gesellschaften sowie der Europäischen Gesellschaft für Endokrine Chirurgie (ESES) zusammensetzt. Ziele des EUROCRINE®-Registers sind die Reduktion von Morbidität und Mortalität aufgrund von Erkrankungen des endokrinen Systems, welches durch den internationalen Vergleich der angewandten Therapiestrategien angestrebt wird [1]. Die sich an EUROCRINE® beteiligenden Kliniken sind primär Zentren, die aktiv die Entscheidung trafen, ihre Ergebnisse und chirurgische Herangehensweise zur Behandlung von Erkrankungen des endokrinen Systems mit anderen europäischen Zentren zu teilen, um eine Optimierung der chirurgischen Versorgung zu erzielen. Daher sind Auswertungen des Registers nicht repräsentativ für die allgemeine Krankenversorgung, sondern spiegeln bereits die Versorgung in Zentren mit endokrin-chirurgischer Spezialisierung wider, wobei für die Teilnahme an EUROCRINE® keine Mindestanzahl spezifischer Operationen gefordert wird.

Europaweit wurden über EUROCRINE® bis zum Jahr 2019 insgesamt 2724 Operationen an Nebennieren durch 119 Kliniken aus 15 Ländern dokumentiert. Weitere Operationen, die mithilfe von EUROCRINE® registriert werden, betreffen die Schilddrüse, Nebenschilddrüsen, neuroendokrine Tumoren des Verdauungstrakts oder aber extraadrenale Paraganglien. Es werden somit auf umfassende Weise alle Gebiete der endokrinen Chirurgie abgedeckt, welche bislang durch andere, nationale Register nicht dokumentiert werden konnten. Über die systematische Dokumentation endokrin-chirurgischer Operationen hinaus bietet das Register zudem die Möglichkeit, Indikationen, präoperative Diagnostik, perioperatives Management sowie umfangreiche postoperative Follow-up-Untersuchungen festzuhalten. Die Datenbank ermöglicht eine selektive Auswertung der Ergebnisse auf Klinik- und Landesebene oder beispielsweise – wie in der vorliegenden Arbeit – auf Ebene der deutschsprachigen EUROCRINE®-Länder: Deutschland, Österreich und Schweiz. Ziel dieser Arbeit war es, eine deskriptive Analyse der bislang in EUROCRINE® in den Jahren 2015 bis 2019 dokumentierten Nebenniereneingriffen der deutschsprachigen EUROCRINE®-Länder (bzw. der überwiegend in der chirurgischen Arbeitsgemeinschaft Endokrinologie (CAEK) der Deutschen Gesellschaft für Allgemein- und Viszeralchirurgie (DGAV) aktiven Kliniken) – im Sinne einer homogenen Versorgungsstruktur – anzufertigen, welche einer ersten Bestandsaufnahme dient und Verbesserungspotenziale für detailliertere Analysen in naher Zukunft eröffnet. Das äußerst flexible Register bietet diesbezüglich Möglichkeiten, einzelne Organmodule entsprechend der sich ergebenden Fragestellungen weiterzuentwickeln und zu überarbeiten.

## Methodik

Die in den Jahren 2015 bis 2019 (Januar 2015 bis September 2019) in Deutschland, Österreich und der Schweiz dokumentierten Nebenniereneingriffe wurden aus dem EUROCRINE®-Register extrahiert. Es erfolgte eine deskriptive Analyse der Operationsindikationen und präoperativen Diagnostik, der angewandten Operationsstrategie (Resektionsausmaß, Resektionstechnik), der zugrunde liegenden histologischen Entitäten und der postoperativen Komplikationsrate. Der exportierte Datensatz wurde unter Verwendung von Microsoft Excel (Microsoft Corporation, Redmond, USA) einer Plausibilitätsprüfung unterzogen. Eine Analyse der Daten sowie graphische Darstellungen wurden mithilfe von Microsoft Excel und Microsoft PowerBI (Microsoft Corporation, Redmond, USA) realisiert. Die Ergebnisse wurden vor dem Hintergrund der aktuellen S2k-Leitlinien der CAEK [2] sowie internationaler Empfehlungen wie beispielsweise der ESES und des European Network for the Study of Adrenal Tumors (ENSAT) diskutiert.

## Ergebnisse

In den 21 Kliniken des deutschsprachigen EUROCRINE®-Gebiets wurden insgesamt 658 Nebennierenoperationen durchgeführt (Tab. 1). 55,9 % der Eingriffe erfolgten bei weiblichen Patienten. Die dokumentierten Eingriffe betrafen in 323 Fällen die linke Nebenniere, in 286 die rechte und in 45 Fällen beide Seiten (4 Fälle ohne Seitenangabe).

**Table 1:** 

	**EUROCRINE® 2015–2019**			
	**Deutschland**	**Österreich**	**Schweiz**	**Gesamt**
**Nebenniereneingriffe gesamt (*n*; %**^**a**^**)**	287; 43,6	139; 21,1	232; 35,3	658; 100
**Kliniken (*n***)	7	3	11	21
**Patientengeschlecht weiblich (*n*; %**^**b**^**)**	174; 60,6	79; 56,8	115; 49,6	368; 55,9
**Patientenalter (Jahre) [Median** **±** **Standardabweichung]**	55 ± 14	54 ± 15	55 ± 15	55 ± 15
**Nebenniereneingriff rechts (*n*; %**^**b**^**)**	131; 45,6	57; 41,0	98; 42,2	286; 43,5
**Nebenniereneingriff links (*n*; %**^**b**^**)**	140; 48,8	72; 51,8	111; 47,8	323; 49,1
**Nebenniereneingriff beidseits (*n*; %**^**b**^**)**	14; 4,9	10; 7,2	21; 9,1	45; 6,8
**Nebenniereneingriff ohne Seitenangabe (*n*; %**^**b**^**)**	2; 0,7	0; 0	2; 0,9	4; 0,6
**Ausmaß Nebenniereneingriffe und Resektionstechniken**				
*Nebenniereneingriffe gesamt *(*n*; %^b^)	287; 100	139; 100	232; 100	658; 100
Laparoskopisch (*n*; %^b^)	188; 65,5	32; 23,0	167; 72,0	387; 58,8
Retroperitoneoskopisch (*n*; %^b^)	22; 7,7	82; 59,0	8; 3,4	112; 17,0
Robotisch assistiert (*n*; %^b^)	0; 0	2; 1,4	1; 0,4	3; 0,5
Minimal-invasiv [nicht spezifiziert] (*n*; %^b^)	8; 2,8	5; 3,6	7; 3,0	20; 3,0
Offen (*n*; %^b^)	53; 18,5	18; 12,9	48; 20,7	119; 18,1
Technik nicht angegeben (*n*; %^b^)	16; 5,6	0; 0	1; 0,4	17; 2,6
Konversion bei minimal-invasiven Verfahren (*n*; %^c^)	11; 5,0	11; 9,1	11; 6,0	33; 6,3
*Unilaterale Adrenalektomie *(*n*; %^b^)	248; 86,4	124; 89,2	214; 92,2	586; 89,1
Laparoskopisch (*n*)	177	24	160	361
Retroperitoneoskopisch (*n*)	21	78	7	106
Robotisch assistiert (*n*)	0	1	1	2
Minimal-invasiv [nicht spezifiziert] (*n*)	6	5	4	15
Offen (*n*)	43	16	41	100
Technik nicht angegeben (*n*)	1	0	1	2
*Bilaterale Adrenalektomie *(*n*; %^b^)	8; 2,8	5; 3,6	7; 3,0	20; 3,0
Laparoskopisch (*n*)	6	4	3	13
Retroperitoneoskopisch (*n*)	0	1	1	2
Offen (*n*)	2	0	3	5
*Nebennierenbiopsie *(*n*; %^b^)	2; 0,7	4; 2,9	3; 1,3	9; 1,4
Laparoskopisch (*n*)	2	1	2	5
Retroperitoneoskopisch (*n*)	0	2	0	2
Robotisch assistiert (*n*)	0	1	0	1
Offen (*n*)	0	0	1	1
*Inzision der Nebennieren *(*n*; %^b^)	0; 0	1; 0,7	0; 0	1; 0,2
Laparoskopisch (*n*)	0	1	0	1
*Andere Nebenniereneingriffe *(*n*; %^b^)	29; 10,1	5; 3,5	7; 3,0	41; 6,2
Laparoskopisch (*n*)	3	2	1	6
Retroperitoneoskopisch (*n*)	1	1	0	2
Minimal-invasiv (nicht spezifiziert) (*n*)	1	0	1	2
Offen (*n*)	9	2	5	16
Andere Techniken (*n*)	15	0	0	15
**Histologie**				
Nebennierenadenom (*n*; %^a^)	131; 48,7	57; 21,2	81; 30,1	269; 100
Phäochromozytom (*n*; %^a^)	71; 46,1	35; 22,7	48; 31,2	154; 100
Nebennierenmetastase anderer Primärtumoren (*n*; %^a^)	18; 32,7	3; 5,5	34; 61,8	55; 100
Nebennierenrindenkarzinom (*n*; %^a^)	22; 61,2	7; 19,4	7; 19,4	36; 100
Nebennierenrindenhyperplasie (*n*; %^a^)	11; 35,5	5; 16,1	15; 48,4	31; 100
Myelolipom (*n*; %^a^)	7; 38,8	1; 5,6	10; 55,6	18; 100
Nebennierenzyste (*n*; %^a^)	3; 37,5	2; 25,0	3; 37,5	8; 100
Lymphom Lokalisation Nebenniere (*n*; %^a^)	0; 0	1; 33,3	2; 66,7	3; 100
Nebennierenmarkhyperplasie (*n*; %^a^)	1; 50,0	1; 50,0	0; 0	2; 100
Hämangiosarkom (*n*; %^a^)	0; 0	1; 50,0	1; 50,0	2; 100
Andere Diagnosen (*n*; %^a^)	23; 28,7	26; 32,5	31; 38,8	80; 100
**Komplikationen**				
*Dokumentierte Komplikationen *(*n*; %^b^)	18; 6,3	5; 3,6	40; 17,2	63; 9,6
Dindo-Clavien Grad 1 (*n*; %^b^)	3; 1,1	1; 0,7	21; 9,1	25; 3,8
Dindo-Clavien Grad 2 (*n*; %^b^)	4; 1,4	3; 2,2	6; 2,6	13; 2,0
Dindo-Clavien Grad 3a (*n*; %^b^)	3; 1,1	0; 0	5; 2,1	8; 1,2
Dindo-Clavien Grad 3b (*n*; %^b^)	4; 1,4	1; 0,7	5; 2,1	10; 1,5
Dindo-Clavien Grad 4b (*n*; %^b^)	1; 0,3	0; 0	3; 1,3	4; 0,6
Dindo-Clavien Grad 5 (*n*; %)^b^	3; 1,1	0; 0	0; 0	3; 0,5

*n* = absolute Anzahl

^a^Prozent von spaltenspezifischem Gesamtwert

^b^Prozent von landesspezifischer Anzahl Nebenniereneingriffe

^c^Prozent von landesspezifischer Anzahl minimal-invasiver Nebenniereneingriffe

### Indikationen zur Nebennierenoperation und präoperative Diagnostik

In 43,9 % (289 von 658 Fällen) handelte es sich um Nebennierentumoren, die initial als Inzidentalome auffällig geworden waren. Der Hauptanteil der Fälle, 55,5 % (365/658), wurde jedoch aufgrund einer Hormonsekretion mit assoziierter Symptomatik diagnostiziert. In 13,5 % (39/658) wurde eine genetische Prädisposition für die Ausbildung von Nebennierentumoren dokumentiert. Als hereditäre Erkrankungen wurden MEN1 (2 Fälle), MEN2A (22 Fälle), MEN2B (3 Fälle), SDHB (1 Fall) und PRKAR1A (2 Fälle) angegeben.

Im Rahmen der präoperativen endokrinologischen Diagnostik wurde zur Abklärung eines möglichen Phäochromozytoms in 63 % (415/658) eine Bestimmung des Metanephrins und in 61,7 % (406/658) des Normetanephrins im Plasma durchgeführt. Die Bestimmung der Katecholaminmetaboliten Epinephrin und Norepinephrin im 24-Stunden-Sammelurin erfolgte in 55,8 % (367/658). Ein Dexamethasonhemmtest zum Ausschluss eines Cushing-Syndroms wurde in 43,6 % (287/658) durchgeführt, wobei das Ergebnis in 82 Fällen auf das Vorliegen eines solchen hinwies. Zur endokrinologischen Abklärung eines Hyperaldosteronismus wurde in 60,5 % (398/658) der Renin-Aldosteron-Quotient bestimmt und ein erhöhter Quotient in 150 dieser Fälle ermittelt. In 40,3 % (265/658) wurde eine Dehydroepiandrosteron-Sulfat(DHEAS)-Bestimmung im Serum erhoben. Hiervon lagen in 23 Fällen pathologisch erhöhte Werte vor. Eine Untersuchung mittels selektiven Venenkatheters zur Lateralisationsdiagnostik hormonaktiver Nebennierentumoren erfolgte in 52 Fällen. Eine Computertomographie (CT) lag präoperativ in 31,2 % (205/658) vor. In 47,1 % (310/658) wurde präoperativ eine Magnetresonanztomographie (MRT) durchgeführt. In 40 Fällen lagen sowohl eine präoperative MRT und eine präoperative CT vor. In 22 Fällen erfolgten zusätzlich funktionelle Bildgebungen mittels F‑DOPA-Positronenemissionstomographie(PET), 6 FDG-PET-CT-Untersuchungen und 3 DOTATOC-PET-CT-Untersuchungen. Aufgrund der präoperativen radiologischen Bildgebung (Morphologie, Größenzunahme, positiver PET-Befund) wurden maligne Nebennierentumoren in 31,3 % (206/658) vermutet. Histologische Ergebnisse einer präoperativen Nebennierenbiopsie lagen in 15,2 % (100/658) vor, in 13 Fällen mit dem Nachweis von Malignität.

### Angewandte Resektionstechniken

**Figure 1:**
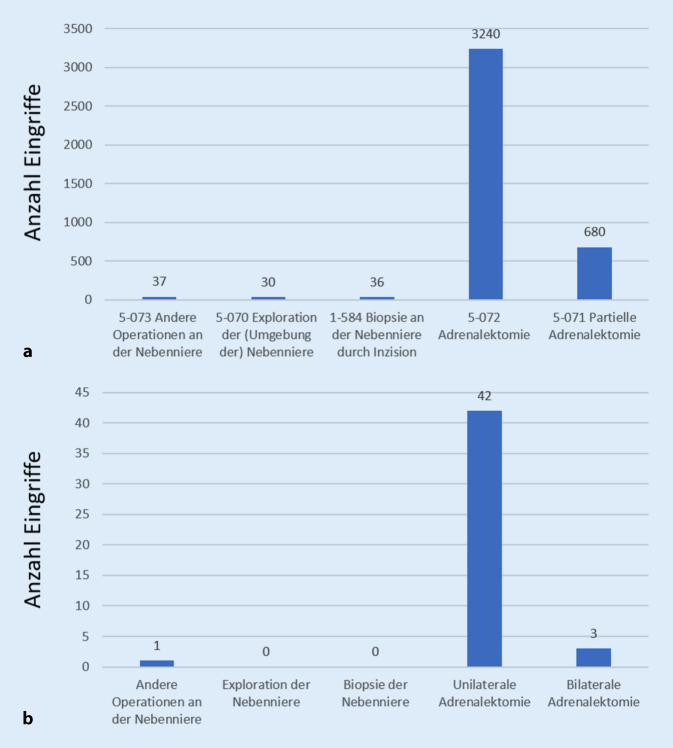

Die überwiegend angewandte Resektionstechnik stellte die laparoskopische Technik dar (387 Eingriffe, 58,8 % aller Nebenniereneingriffe). Offene transabdominelle Nebennierenresektionen erfolgten an zweithäufigster Stelle (119 Eingriffe, 18,1 % aller Nebenniereneingriffe), gefolgt von retroperitoneoskopischen Eingriffen (112 Eingriffe, 17 % aller Nebenniereneingriffe). Eine Konversion wurde bei minimal-invasiv begonnenen Operationen in 6,3 % notwendig. Die mit 89,1 % am häufigsten durchgeführte Operation stellte die unilaterale Adrenalektomie dar (Tab. 1). Ein exemplarischer Vergleich der in Deutschland im Jahr 2016 in EUROCRINE® dokumentierten Operationen mit den durch das Statistische Bundesamt dokumentierten Operationen- und Prozedurenschlüssel (OPS-Codes) desselben Beobachtungszeitraums zeigte, dass die primär angewandte Resektionsstrategie in beiden Fällen die unilaterale Adrenalektomie darstellte (Abb. 1). Der direkte Vergleich illustrierte jedoch zudem, dass über EUROCRINE® bislang lediglich ein Bruchteil der insgesamt in Deutschland durchgeführten Nebenniereneingriffe abgebildet wurde. Aus Österreich und der Schweiz lagen keine entsprechenden Daten vor.

Die in den Jahren 2015 bis 2019 in den deutschsprachigen EUROCRINE®-Ländern dokumentierten unilateralen Adrenalektomien erfolgten zu 61,6 % laparoskopisch (361 Operationen, Tab. 1). Weitere 18,1 % der unilateralen Adrenalektomien wurden retroperitoneoskopisch durchgeführt (106 Operationen). Die offen-chirurgische Resektion erfolgte in 17 % (100 Operationen), während robotisch assistierte Operationen selten durchgeführt wurden (2 Operationen). Bilaterale Adrenalektomien stellten 3 % der Gesamteingriffe dar. Diese wurden zu 65 % (13 Operationen) laparoskopisch durchgeführt, während 5 bilaterale Adrenalektomien offen-chirurgisch erfolgten und 2 Operationen über einen retroperitoneoskopischen Zugang ausgeführt wurden (Tab. 1).

### Minimal-invasive Verfahren im deutschsprachigen EUROCRINE®-Gebiet

**Figure 2:**
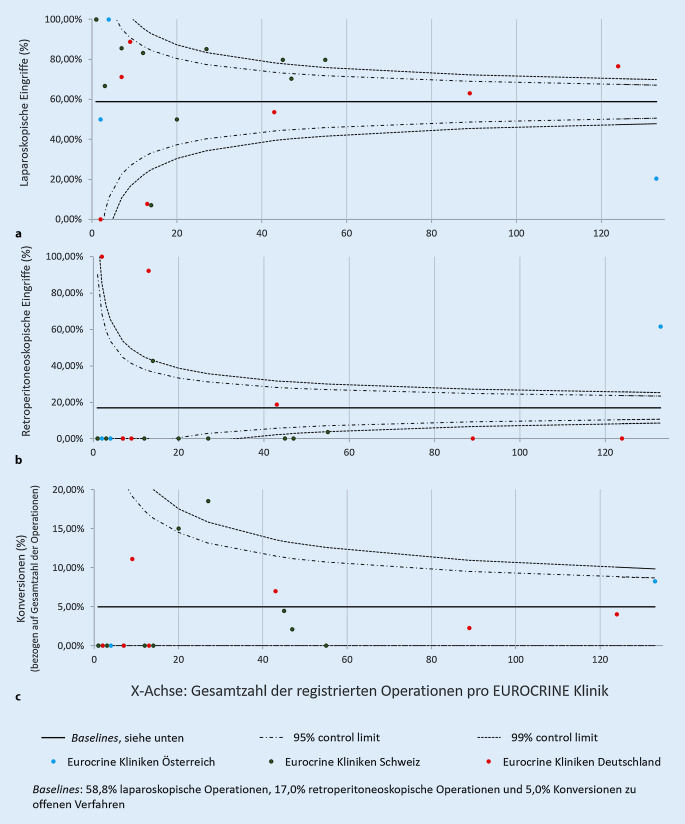

In den an EUROCRINE® teilnehmenden Kliniken in Deutschland wurden 65,5 % der Nebenniereneingriffe von 2015 bis 2019 laparoskopisch durchgeführt. In der Schweiz ließen sich 72 % laparoskopische Operationen verzeichnen. Retroperitoneoskopische Verfahren wurden in 7,7 % bzw. 3,4 % der Operationen angewandt (Tab. 1). In Österreich bestand mit 82 Operationen (59 %) – gegenüber laparoskopischen Eingriffen (32 Operationen, 23 %) – eine Präferenz für retroperitoneoskopische Verfahren (Tab. 1). Die klinikbezogene Darstellung der angewandten minimal-invasiven Techniken (Abb. 2) ergab diesbezüglich, dass die hohe Anzahl retroperitoneoskopischer Operationen innerhalb Österreichs insbesondere einer „High-volume“-Klinik zuzuordnen ist. Dies illustriert den bestehenden Selektionsbias durch die Teilnahme primär endokrin-chirurgisch spezialisierter Zentren an EUROCRINE®. Robotisch assistierte Adrenalektomien (sowohl von trans- als auch von retroperitoneal) wurden ebenfalls durchgeführt, jedoch erfolgten diese Verfahren höchst selten (2 Operationen, durchgeführt bei Nebennierenadenomen, Tab. 1). Eine Konversion zu offen-chirurgischen Verfahren wurde bei insgesamt 6,3 % (33/522) minimal-invasiv begonnener Eingriffe dokumentiert.

### Häufigkeitsverteilung histologischer Diagnosen

**Figure 3:**
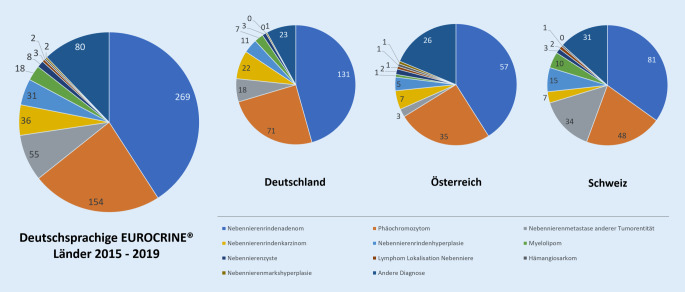

Die in 40,8 % der Gesamtoperationen zugrunde liegende histologische Diagnose war das adrenokortikale Adenom (Tumordurchmesser Median: 28 mm, Range: 4–115 mm, 34 ohne Größenangabe; Tab. 2, Abb. 3). 23,4 % der dokumentierten Nebenniereneingriffe erfolgten aufgrund eines Phäochromozytoms, welches somit die zweithäufigste histologische Diagnose darstellte. In 20,1 % der operierten Phäochromozytome wurde eine genetische Prädisposition dokumentiert (Tab. 3). An dritter Stelle rangierte mit 8,4 % der Fälle die Gruppe der Nebennierenmetastasen anderer maligner Grunderkrankungen, welche eine Resektion erfuhren. Diese Gruppe findet sich somit vor den Nebennierenrindenkarzinomen, die lediglich in 5,5 % der Fälle vorlagen (Abb. 3). Der mediane Tumordurchmesser der operierten Nebennierenkarzinome betrug 100 mm mit einer Spannweite von 13–410 mm (Tab. 4). Lediglich bei 29 der postoperativ festgestellten 36 Nebennierenrindenkarzinome war präoperativ das Vorliegen eines malignen Tumors angenommen worden. Retrospektiv lag in der Gruppe der Nebennierenrindenkarzinome in 38,9 % der Fälle präoperativ eine klinische Symptomatik bedingt durch Hormonsekretion vor (Tab. 4). Innerhalb der insgesamt 206 präoperativ aufgrund radiologischer Befunde als potenziell maligne eingestuften Nebennierentumoren bestätigten sich postoperativ in 48,1 % der Fälle (99/206) maligne Läsionen. Hierunter befanden sich 29 Nebennierenrindenkarzinome und 53 Nebennierenmetastasen anderer bösartiger Grunderkrankungen.

**Table 2:** 

Nebennierenrindenadenome – EUROCRINE® 2015–2019				
	Deutschland	Österreich	Schweiz	Gesamt
**Nebenniereneingriffe gesamt** (*n*; %^a^)	131; 48,7	57; 21,2	81; 30,1	269; 100
**Patientengeschlecht weiblich** (*n*; %^b^)	84; 64,1	39; 68,4	38; 46,9	161; 59,9
**Patientenalter** (Jahre) [Median± Standardabweichung]	55 ± 12	51 ± 14	53 ± 12	53 ± 13
**Präoperative Situation**				
*Klinische Symptome durch Hormonexzess (n; *%^b^)	75; 57,3	39; 68,4	55; 67,9	169; 62,8
Kortisol (Cushing-Syndrom-Patienten)	15	15	9	39
Cushing subklinisch	2	5	1	7
Aldosteron (Conn-Syndrom-Patienten)	56	17	45	118
Androgene	2	1	0	3
Kortisol, Androgene	0	1	0	2
**Ausmaß Nebenniereneingriffe und Resektionstechniken**				
**Alle Nebennierenadenome**				
*Nebenniereneingriffe gesamt *(*n*; %^*b*^)	**131; 48,7**	**57; 21,2**	**81; 30,1**	**269; 100**
Laparoskopisch (*n*)	95	11	69	175
Retroperitoneoskopisch (*n*)	13	41	2	56
Robotisch assistiert (*n*)	0	1	1	2
Minimal-invasiv [nicht spezifiziert] (*n*)	2	2	1	5
Offen (*n*)	14	2	7	23
Technik nicht angegeben (*n*)	7	0	1	8
Konversion bei minimal-invasiven Verfahren (*n*; %^c^)	2; 1,8	3; 5,5	2; 2,7	7; 2,9
*Unilaterale Adrenalektomie *(*n*; %^*b*^)	120; 91,6	52; 91,2	81; 100	253; 94,1
*Bilaterale Adrenalektomie *(*n*; %^*b*^)	0; 0	2; 3,5	0; 0	2; 0,7
*Nebennierenbiopsie *(*n*; %^*b*^)	1; 0,8	2; 3,5	0; 0	3; 1,1
*Andere Nebenniereneingriffe *(*n*; %^b^)	10; 7,6	1; 1,8	0; 0	11; 4,1
**Darunter Conn-Syndrom-Patienten**				
*Nebenniereneingriffe gesamt *(*n*; %^b^)	**56; 42,7**	**17, 29,8**	**45, 55,5**	**118; 43,9**
Laparoskopisch (*n*)	44	3	40	87
Retroperitoneoskopisch (*n*)	7	14	1	22
Robotisch assistiert (*n*)	0	0	1	1
Offen (*n*)	2	0	3	5
Technik nicht angegeben (*n*)	3	0	0	3
Konversion bei minimal-invasiven Verfahren (*n*; %^c^)	0	0	0	0
*Unilaterale Adrenalektomie *(*n*; %^b^)	52	16	45	113
*Nebennierenbiopsie *(*n*; %^b^)	0	1	0	1
*Andere Nebenniereneingriffe *(*n*; %^b^)	4	0	0	4
**Darunter Cushing-Syndrom-Patienten**				
*Nebenniereneingriffe gesamt *(*n*; %^b^)	**15; 11,5**	**15; 26,3**	**9; 11,1**	**39; 14,5**
Laparoskopisch (*n*)	10	4	8	22
Retroperitoneoskopisch (*n*)	2	10	0	12
Minimal-invasiv (nicht spezifiziert) (*n*)	1	0	1	2
Offen (*n*)	0	1	0	1
Technik nicht angegeben (*n*)	2	0	0	2
Konversion bei minimal-invasiven Verfahren (*n*; %^c^)	1; 7,7	1; 7,1	1; 11,1	3; 8,3
*Unilaterale Adrenalektomie *(*n*; %^b^)	12	13	9	34
*Bilaterale Adrenalektomie *(*n*; %^b^)	0	2	0	2
*Andere Nebenniereneingriffe *(*n*; %^b^)	3	0	0	3
**Histologie**				
Tumordurchmesser (mm) [Median, Range]	28, 5–114	30; 6–90	25; 4–115	28, 4–115
(Tumordurchmesser nicht angegeben)	8	22	4	34

*n* = absolute Anzahl

^a^Prozent von spaltenspezifischem Gesamtwert

^b^Prozent von landesspezifischer Anzahl Nebenniereneingriffe

^c^Prozent von landesspezifischer Anzahl minimal-invasiver Nebenniereneingriffe

**Table 3:** 

**Phäochromozytom – EUROCRINE® 2015–2019**				
	**Deutschland**	**Österreich**	**Schweiz**	**Gesamt**
**Nebenniereneingriffe gesamt **(*n*; %^a^)	71; 46,1	35; 22,7	48; 31,2	154; 100
**Patientengeschlecht weiblich **(*n*; %^b^)	43; 60,6	19; 54,3	30; 62,5	92; 59,7
**Patientenalter (Jahre) [Median** **±** **Standardabweichung]**	53 ± 16	52 ± 16	49 ± 17	52 ± 16
**Präoperative Situation**				
*Genetische Mutation *(*n*; %^b^)	18; 25,4	8; 22,9	5; 10,4	31; 20,1
MEN2A	12	6	2	20
MEN2B	2	0	1	3
NF1	2	1	0	3
RET	1	0	0	1
SDHB	0	1	0	1
VHL	1	0	2	3
*Klinische Symptome durch Katecholaminexzess *(*n*; %^b^)	61; 85,9	28; 80,0	43; 89,6	132; 85,7
Medikamentöse Therapie bei Katecholaminexzess (*n*; %^b^)	54; 88,5	27; 96,4	0; 0	81;61,4
**Ausmaß Nebenniereneingriffe und Resektionstechniken**				
*Nebenniereneingriffe gesamt *(*n*; %^b^)	71; 100	35; 100	48; 100	154; 100
Laparoskopisch (*n*)	54	9	36	99
Retroperitoneoskopisch (*n*)	4	19	3	26
Minimal-invasiv (nicht spezifiziert) (*n*)	1	0	0	1
Offen (*n*)	8	7	9	24
Technik nicht angegeben (*n*)	4	0	0	4
Konversion bei minimal-invasiven Verfahren (*n*; %^c^)	2; 3,4	2; 7,1	1; 2,6	5; 4,0
*Unilaterale Adrenalektomie *(*n*; %^b^)	61; 85,9	32; 91,4	46; 95,8	139; 90,3
*Bilaterale Adrenalektomie *(*n*; %^b^)	3; 4,3	0; 0	1; 2,1	4; 2,6
*Nebennierenbiopsie *(*n*; %^b^)	0; 0	2; 5,7	0; 0	2; 1,3
*Andere Nebenniereneingriffe *(*n*; %^b^)	7; 9,9	1;2,9	1; 2,1	9; 5,8
**Histologie**				
Anteil maligner Phäochromozytome (*n*; %^b^)	3; 4,2	2; 5,7	1; 2,0	6; 3,9
Tumordurchmesser (mm) [Median, Range]	38, 13–740	34, 3–130	40, 8–650	38, 3–740
(Tumordurchmesser nicht angegeben)	3	11	2	16

*n* = absolute Anzahl

^a^Prozent von spaltenspezifischem Gesamtwert

^b^Prozent von landesspezifischer Anzahl operierte Phäochromozytome

^c^Prozent von landesspezifischer Anzahl minimal-invasiver Nebenniereneingriffe bei Phäochromozytomen

**Table 4:** 

**Nebennierenrindenkarzinom – EUROCRINE® 2015–2019**				
	**Deutschland**	**Österreich**	**Schweiz**	**Gesamt**
**Nebenniereneingriffe gesamt **(*n*; %^a^)	22; 61,2	7; 19,4	7; 19,4	36; 100
**Patientengeschlecht weiblich **(*n*; %^b^)	14; 63,6	4; 57,1	7; 100	25; 69,4
**Patientenalter (Jahre) [Median** **±** **Standardabweichung]**	58 ± 17	62 ± 19	56 ± 23	58 ± 19
**Präoperative Situation**				
Klinische Symptome durch Hormonexzess (*n*; %^b^)	5; 22,7	4; 57,1	5; 71,4	14; 38,9
Kortisol	3	3	3	9
Aldosteron	0	0	1	1
Androgene	1	1	0	2
Kortisol, Aldosteron	1	0	0	1
Kortisol, Androgene	0	0	1	1
Präoperativ radiologisch Verdacht auf Malignität (*n*; %^b^)	19; 86,4	4; 57,1	6; 85,7	29; 80,6
**Ausmaß Nebenniereneingriffe und Resektionstechniken**				
*Nebenniereneingriffe gesamt *(*n*; %^b^)	22; 100	7; 100	7; 100	36; 100
Laparoskopisch (*n*)	5	0	1	6
Retroperitoneoskopisch (*n*)	0	2	0	2
Minimal-invasiv (nicht spezifiziert) (*n*)	0	2	0	2
Offen (*n*)	17	3	6	26
Konversion bei minimal-invasiven Verfahren (*n*; %^c^)	0; 0	2; 50,0	0; 0	2; 20,0
*Unilaterale Adrenalektomie *(*n*; %^b^)	18; 81,8	7; 100	6; 85,7	31; 86,1
*Andere Nebenniereneingriffe *(*n*; %^b^)	4; 18,2	0; 0	1; 14,3	5; 13,9
**Histologie**				
Tumordurchmesser (mm) [Median, Range]	100, 13–190	90, 40–145	105, 18–410	100, 13–410
(Tumordurchmesser nicht angegeben)	1	2	0	3
**Postoperative Situation**				
*Follow-up (Monate postoperative) [Median, Range]*	0, 0–16	0, 0	1, 0–7	0, 0–16
*Reoperation aufgrund eines Rezidivs*	1	0	0	1
*Onkologische Therapie*	6	0	5	11
Mitotane	5	0	4	9
Mitotane, Chemotherapie^d^	1	0	0	1
Mitotane, Chemotherapie^d^, Strahlentherapie	0	0	1	1

*n* = absolute Anzahl

^a^Prozent von spaltenspezifischem Gesamtwert

^b^Prozent von landesspezifischer Anzahl operierte Nebennierenrindenkarzinome

^c^Prozent von landesspezifischer Anzahl minimal-invasiver Nebenniereneingriffe bei Nebennierenrindenkarzinomen

^d^Etoposide-Doxorubicin-Cisplatin/Streptozocin

### Histologische Hauptdiagnosen und Resektionsverfahren

Nebennierenrindenadenome wurden überwiegend durch minimal-invasive Verfahren (laparoskopisch > retroperitoneoskopisch) reseziert (Tab. 2). Das in erster Linie umgesetzte Resektionsverfahren stellte in allen untersuchten Ländern die unilaterale Adrenalektomie dar (Tab. 2). Phäochromozytome wurden in 81,8 % über minimal-invasive Verfahren operiert, hiervon 64,2 % laparoskopisch und 16,8 % retroperitoneoskopisch (Tab. 3). Der mediane Tumordurchmesser minimal-invasiv operierter Phäochromozytome lag bei 38 mm (Range: 3–95 mm, 12 ohne Größenangabe). 4 % dieser Eingriffe erforderten eine Konversion zur offenen Resektion. Der mediane Tumordurchmesser offen-chirurgisch resezierter Phäochromozytome lag bei 60 mm (Range: 5–180 mm, 4 ohne Größenangabe). Primär erfolgten bei der Diagnose „Phäochromozytom“ unilaterale Adrenalektomien (90,3 %, Tab. 3). Die Resektion von Nebennierenrindenkarzinomen wurde in 27,8 % minimal-invasiv begonnen (einschließlich 16,7 % laparoskopische und 5,6 % retroperitoneoskopische Operationen, Tab. 4). Eine Konversion wurde in 20 % (2/10 Fälle) der minimal-invasiv begonnenen Eingriffe notwendig. Die Betrachtung einer Subgruppe von Nebennierenkarzinomen mit einem Durchmesser >5 cm (26 Fälle) zeigte, dass in diesen Fällen in 11,5 % (3/26) eine laparoskopische Resektionsstrategie gewählt wurde. Diese Operationen erforderten jedoch in 2 der 3 Fälle eine Konversion zur offenen Resektion. In einem dieser Fälle, bei dem ein 8 cm messendes Nebennierenrindenkarzinom zugrunde lag, war initial keine Malignität vermutet worden. Bei dem zweiten Fall handelte es sich um ein 14,5 cm messendes ACC. Hierbei war präoperativ bereits ein maligner Befund angenommen worden, jedoch musste aus nicht angegebenen Gründen eine Konversion erfolgen. Nebennierenkarzinome mit einem Durchmesser >6 cm wurden sonst ausschließlich offen-chirurgisch reseziert (23 Fälle). Nebennierenmetastasen wurden in 65,5 % über minimal-invasive Verfahren operiert (Tab. 5). Der mediane Tumordurchmesser betrug 35 mm (Range: 6‑150, Tab. 5). Eine Konversion erfolgte in 19,4 % der minimal-invasiv operierten Fälle.

**Table 5:** 

**Nebennierenmetastasen anderer Primärtumoren – EUROCRINE® 2015–2019**				
	**Deutschland**	**Österreich**	**Schweiz**	**Gesamt**
**Nebenniereneingriffe gesamt (*n*; %**^**a**^**)**	18; 32,7	3; 5,5	34; 61,8	55; 100
**Patientengeschlecht weiblich (*n*; %**^**b**^**)**	6; 33,3	2; 66,7	8; 23,5	16; 29,1
**Patientenalter (Jahre) [Median± Standardabweichung]**	59 ± 10	64±2	62±9	61±9
**Ausmaß Nebenniereneingriffe und Resektionstechniken**				
*Nebenniereneingriffe gesamt *(*n*; %^b^)	18; 100	3; 100	34; 100	55; 100
Laparoskopisch (*n*)	8	0	20	28
Retroperitoneoskopisch (*n*)	0	2	1	3
Minimal-invasiv [nicht spezifiziert] (*n*)	2	0	3	5
Offen (*n*)	6	1	10	17
Technik nicht angegeben (*n*)	2	0	0	2
Konversion bei minimal-invasiven Verfahren (*n*; %^c^)	3; 30,0	1; 50,0	3; 12,5	7; 19,4
*Unilaterale Adrenalektomie *(*n*; %^b^)	14; 77,8	3; 100	31; 91,2	48; 87,3
*Bilaterale Adrenalektomie *(*n*; %^b^)	2; 11,1	0; 0	1; 2,9	3; 5,5
*Nebennierenbiopsie *(*n*; %^b^)	0; 0	0; 0	2; 5,9	2; 3,6
*Andere Nebenniereneingriffe *(*n*; %^b^)	2; 11,1	0; 0	0; 0	2; 3,6
**Histologie**				
Tumordurchmesser (mm) [Median ± Range]	35; 7–120	56, 35–75	38, 6–150	35, 6–150
(Tumordurchmesser nicht angegeben)	1	1	4	6
**Postoperative Situation**				
Letztes Follow-up (Monate) [Median, Range]	0, 0–6	0, 0–2	0, 0–6	0, 0–6
Reoperation aufgrund eines Rezidivs	0	0	2	2

*n* = absolute Anzahl

^a^Prozent von spaltenspezifischem Gesamtwert

^b^Prozent von landesspezifischer Anzahl operierte Nebennierenmetastasen

^c^Prozent von landesspezifischer Anzahl minimal-invasiver Nebenniereneingriffe bei Nebennierenmetastasen

### Postoperatives Follow-up

**Figure 4:**
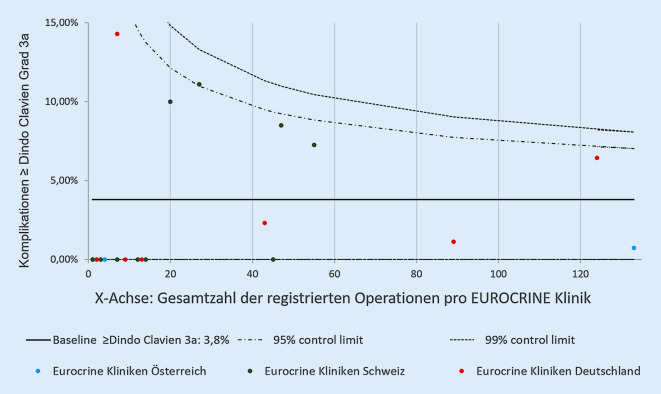

Die allgemeine Komplikationsrate des betrachteten Kollektivs lag bei 9,6 %. Hauptsächlich waren diese Grad 1 (3,8 %) und Grad 2 (2 %) nach Dindo-Clavien-Klassifikation [3] zuzuordnen. Es wurden jedoch auch Komplikationen Grad 3a (1,2 %), Grad 3b (1,5 %) und Grad 4a (0,6 %) beobachtet (Abb. 4). Drei Patienten verstarben im frühpostoperativen Verlauf (Tab. 1).

Bei der Diagnose Nebennierenrindenkarzinom wurde in 30,6 % eine onkologische Nachbehandlung dokumentiert (Tab. 5). 25 % der Patienten erfuhren eine Behandlung mit Mitotane [4]. Jeweils 2,8 % der Patienten wurden mit Mitotane in Kombination mit einer Chemotherapie (Etoposid, Doxorubicin, Cisplatin/Streptozocin; [5]) bzw. mit einer zusätzlichen Bestrahlung therapiert (Tab. 5).

Aufgrund der bislang kurzen Zeit der Verfügbarkeit des EUROCRINE®-Registers sind keine Follow-up-Daten zu den dokumentierten Operationen vorhanden.

## Diskussion

Aus dem Vergleich mit der durch das Statistische Bundesamt veröffentlichten Dokumentation der Operationen- und Prozedurenschlüssel (OPS-Codes) des Jahres 2016 geht hervor, dass in diesem Zeitraum in Deutschland insgesamt 4023 Eingriffe an Nebennieren erfolgten (Abb. 1). Die in diesem Zeitraum in Deutschland über EUROCRINE® dokumentierten Nebenniereneingriffe stellen lediglich einen Bruchteil dieser Operation dar, sodass davon ausgegangen werden muss, dass die vorliegende Arbeit nicht die breite Versorgungsrealität Deutschlands oder der zusätzlich betrachteten Länder Österreich und Schweiz widerspiegelt. Aufgrund der freiwilligen Teilnahme an EUROCRINE® sind Kliniken mit ausgewiesenem endokrin-chirurgischem Schwerpunkt in dieser Stichprobe überrepräsentiert. Mit zunehmender Anzahl teilnehmender Kliniken kann in Zukunft ein repräsentativeres Bild der tatsächlichen klinischen Versorgungssituation in Relation mit den geltenden Leitlinien sowie im Vergleich mit internationalen Forschungsergebnissen ermöglicht werden.

Auffällig an den dargelegten ersten Ergebnissen der in den Jahren 2015 bis 2019 dokumentierten Nebennierenoperationen des EUROCRINE®-Registers war zunächst, dass eine überraschend hohe Anzahl von Nebennierenmetastasen anderer Tumorentitäten enthalten war. Nebennierenmetastasen stellten nach dem Nebennierenrindenadenom und dem Phäochromozytom die dritthäufigste histologische Hauptdiagnose des Kollektivs dar (Abb. 3). Da die genaue Indikation zur Durchführung der Resektion bislang nicht eindeutig im EUROCRINE®-Register dokumentiert wurde, muss an dieser Stelle davon ausgegangen werden, dass diese Resektionen größtenteils im Rahmen der Umsetzung individuell geplanter, onkologischer Konzepte zur Therapie der vorliegenden Grunderkrankungen erfolgten. Die länderspezifische Betrachtung zeigte, dass über 60 % der dokumentierten Operationen bei Nebennierenmetastasen in der Schweiz stattfanden (Tab. 1). Auch wenn epidemiologisch betrachtet die Gruppe der Nebennierenmetastasen nach dem Nebennierenrindenadenom die häufigste Ursache von Nebennierenraumforderungen darstellt [6], wird die Indikation zu deren Operation nicht uneingeschränkt getroffen. Da das Gesamtüberleben unter anderem deutlich durch die maligne Grunderkrankung beeinflusst wird, konnte bislang ein genereller onkologischer Vorteil durch eine Resektion von Nebennierenmetastasen nicht sicher nachgewiesen werden [6, 7]. Nebennierenmetastasen werden beispielsweise bei nichtkleinzelligen Bronchialkarzinomen, malignen Melanomen, Mammakarzinom oder hepatozellulären Karzinomen beobachtet [6, 8, 9]. Nach Empfehlung der CAEK kann bei Nebennierenmetastasen im Rahmen individueller Konzepte eine Resektion indiziert sein. Insbesondere bei isolierten Nebennierenmetastasen bei metachroner Erkrankung kann eine Adrenalektomie das Gesamtüberleben günstig beeinflussen [7, 8]. Folglich sollte ein interdisziplinäres Tumorboard das gewählte Therapiekonzept tragen [2]. Eine Resektion kann bei vorhandener chirurgischer Expertise und dem Vorliegen lokal begrenzter Läsionen ohne Infiltration von Nachbarstrukturen minimal-invasiv erfolgen (R12 CAEK; [2, 10, 11]). Die Nebennierenmetastasen im betrachteten Kollektiv wurden in 65,5 % der Fälle minimal-invasiv reseziert. Eine Korrelation zwischen Klinikvolumen und der Tendenz zur Resektion von Nebennierenmetastasen bestand im vorliegenden Kollektiv nicht.

**Figure 5:**
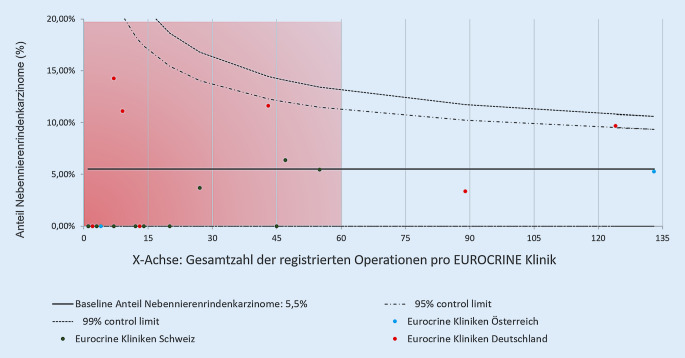

Ein weiterer überraschender Aspekt der aktuellen Untersuchung war die Häufigkeit der minimal-invasiv geplanten Operationen bei Nebennierenrindenkarzinomen und die damit verbundene Häufigkeit einer Konversion zur offenen Resektion. Nach den aktuellen Leitlinien der CAEK sollten nichtfunktionelle Nebennierentumoren mit einem Größendurchmesser von ≥6 cm aufgrund des Malignitätsrisikos offen-chirurgisch reseziert werden (R4 CAEK; [2]). Zudem existiert die klare Empfehlung der offenen Resektion bei präoperativen Hinweisen auf das Vorliegen eines Nebennierenrindenkarzinoms (R10 CAEK). Bei ausreichender chirurgischer Expertise kann jedoch alternativ bei Tumoren <6 cm eine minimal-invasive Resektion erwogen werden [2]. Die ESES/ENSAT-Leitlinien empfehlen ebenfalls ein primär offenes chirurgisches Vorgehen bei bestehender Diagnose eines Nebennierenrindenkarzinoms (R10 ESES/ENSAT), jedoch wird auch hier eine Möglichkeit der laparoskopischen Resektion bei vorhandener Expertise und lokal begrenzten Befunden eingeräumt (R12, R13 ESES/ENSAT; [12, 13]). In diesem Sinne wurden in der aktuellen Auswertung des EUROCRINE®-Registers 27,8 % minimal-invasive Eingriffe zur Resektion von Nebennierenrindenkarzinomen registriert, welche mit einer Konversionsrate von 20 % assoziiert waren. In 72,2 % der operierten Nebennierenkarzinome lagen Tumordurchmesser von >5 cm vor. In 11,5 % dieser Fälle wurde eine laparoskopische Resektionsstrategie geplant und begonnen. Diese Operationen erforderten jedoch in 66,7 % eine Konversion. Die American Association of Endocrinologists gibt in ihren Empfehlungen zum Management von Inzidentalomen zu bedenken, dass Tumoren, welche initial nicht als Nebennierenrindenkarzinom erkannt wurden und sich intraoperativ als solches darstellen, eine Konversion von einer minimal-invasiven Adrenalektomie zu einer offenen Resektion rechtfertigen, da durch eine offen-chirurgische Versorgung das Risiko eines frühen Rezidivs und einer peritonealen Aussaat reduziert werden könne [4, 14–17]. Durch die bereits ähnliche Patientenlagerung bei der laparoskopischen Adrenalektomie könne im Vergleich zu retroperitoneoskopischen Verfahren eine raschere Konversion ermöglicht werden, welches sich vorteilhaft auswirke [18]. In Bezug auf die postoperative Komplikationsrate konnten Thompson et al. jedoch in einer multivariaten Analyse von Adrenalektomien innerhalb des Scandinavian Quality Register for Thyroid, Parathyroid and Adrenal Surgery (SQRTPA) zeigen, dass eine Konversion von minimal-invasiven Verfahren zur offen-chirurgischen Versorgung mit einem erhöhten Komplikationsrisiko assoziiert war, wohingegen der Faktor einer primär offen-chirurgischen Operation sich nicht signifikant auf das Auftreten postoperativer Komplikationen auswirkte [19]. Dies unterstreicht die Bedeutung der präoperativen Diagnostik zur adäquaten, befundadaptierten Operationsplanung und Verfahrenswahl bei Nebennierenraumforderungen. Zudem wird durch die ESES/ENSAT 2017 empfohlen, dass Operationen bei Nebennierenkarzinomen lediglich in Zentren erfolgen sollten, denen Chirurgen ein Eingriffsvolumen von mindestens 15 Nebennierenringriffe pro Jahr vorweisen können (R 7; [12]). Ziel der Empfehlung ist es, bei geringen Komplikationsraten ein optimales onkologisches Ergebnis zu ermöglichen. In der vorliegenden Analyse zeigte sich jedoch, dass einige Kliniken über den nahezu vierjährigen Zeitraum des Bestehens des EUROCRINE®-Registers eine Gesamtzahl von unter 60 Nebenniereneingriffen aufwiesen und dennoch Operationen bei Nebennierenrindenkarzinomen durchführten (Abb. 5). Da aus der aktuellen Auswertung jedoch nicht hervorgeht, zu welchem Zeitpunkt nach der Etablierung des EUROCRINE®-Registers die jeweiligen Kliniken an der Dokumentation teilnahmen, kann hier eine deutliche Unterschätzung der tatsächlich pro Jahr durchgeführten Nebennierenoperationen zugrunde liegen. Der Hauptteil der Nebennierenrindenkarzinome des betrachteten Kollektivs wurde jedoch Leitliniengerecht an „High-volume“-Zentren durchgeführt. Weiterhin kann, insbesondere bei einer geringen Tumorgröße von unter 6 cm, nicht in jedem Fall präoperativ sicher zwischen malignen und benignen Befunden unterschieden werden, sodass Nebennierenrindenkarzinome als Zufallsbefunde – unabhängig von Eingriffsvolumina – möglich sind.

In 20,1 % aller Phäochromozytome des Kollektivs wurde eine hereditäre Grunderkrankung bzw. Mutation dokumentiert. Während in Deutschland und Österreich in über 20 % eine genetische Prädisposition erfasst wurde, lag die Rate in der Schweiz lediglich bei 10 % (Tab. 2). Nach Angaben der Literatur kann jedoch bei über einem Viertel der Phäochromozytompatienten eine genetische Prädisposition erwartet werden [4]. Da 37 % der in der Schweiz operierten Phäochromozytompatienten (18/48) ein Lebensalter <45 Jahren aufwiesen, kann eine hereditäre Genese zumindest bei einem Teil dieser Patienten als wahrscheinlich angenommen werden. Bei Phäochromozytomen spielen, insbesondere bei Patienten mit einer genetischen Prädisposition, nebennierenparenchymerhaltende Eingriffe eine größere Rolle. Aktuell kann jedoch die „Cortical-sparing“-Variante [20] einer Adrenalektomie nicht als solche im EUROCRINE®-Register angegeben werden. Diese Resektionen finden sich unter „andere Nebenniereneingriffe“ wieder (Tab. 3). Abhängig vom präoperativ bestimmten Tumordurchmesser wurden in den betrachteten EUROCRINE®-Ländern präferiert minimal-invasive Verfahren (einschließlich 64,2 % laparoskopisch, 16,8 % retroperitoneoskopisch) angewandt. Die bereits beschriebene Präferenz für die laparoskopische Technik bei Nebenniereneingriffen zeichnete sich ebenso bei Patienten mit Conn- bzw. Cushing-Syndrom ab, wobei die in erster Linie erfolgte Resektionstechnik für beide Untergruppen die unilaterale Adrenalektomie darstellte. Lediglich in Österreich bestand eine Präferenz für retroperitoneoskopisch durchgeführte Adrenalektomien, wobei das Ergebnis insbesondere durch eine „High-volume“-Klinik innerhalb des Landes beeinflusst wurde. Die CAEK empfiehlt, dass primäre Nebennierentumoren ≤6 cm in Abwesenheit von Malignitätshinweisen minimal-invasiv reseziert werden sollten (R2 CAEK; [2]). Nach Empfehlung der CAEK sind die retroperitoneoskopische und laparoskopische Technik als gleichwertig anzusehen. Die Wahl des Resektionsverfahren wird der Präferenz des Operateurs freigestellt (R3 CAEK; [2]). Die Analyse der Resektionsverfahren aller Nebenniereneingriffe in den teilnehmenden EUROCRINE®-Kliniken belegt, dass sowohl in Deutschland als auch in Österreich und der Schweiz beide der zuvor genannten minimal-invasiven Verfahren durchgeführt wurden. Jedoch zeichnete sich klar ab, dass das laparoskopische Vorgehen weiterhin die primäre Wahl des Großteils der Operateure im deutschsprachigen EUROCRINE®-Gebiet darstellte, mit Ausnahme weniger Kliniken (Abb. 2). Die robotisch assistierte Adrenalektomie wurde ebenfalls durchgeführt, jedoch wurde dieses Verfahren im untersuchten Kollektiv deutlich seltener angewandt. Wie aus der Literaturrecherche, die den Leitlinien der CAEK zugrunde liegt, hervorgeht, ist die Anwendung robotisch assistierter Verfahren bislang ohne Vorteil gegenüber anderen minimal-invasiven Verfahren (R6 CAEK; [2]), was einen Grund für die Seltenheit der Durchführung darstellen kann. Im Kontrast hierzu hatte eine im Jahr 2017 publizierte Analyse des Skandinavischen Qualitätsregisters SQRTPA ergeben, dass die Hälfte der minimal-invasiven Nebennierenoperationen in Schweden bereits robotisch assistiert erfolgte [19]. Hinsichtlich der Konversionsrate robotisch assistierter Eingriffe hatte sich in einer multivariaten Analyse kein Nachteil gegenüber den minimal-invasiven Verfahren ergeben [19].

## Limitationen

Eine Limitation der Auswertung des Registers stellt zum aktuellen Zeitpunkt die Unvollständigkeit der Follow-up-Einträge dar. Dies betrifft beispielsweise die histologische Dokumentation der Nebennierenrindenkarzinome (TNM-Klassifikation und die durch die ESES/ENSAT angestrebte Dokumentation des Ki67-Index [12]). Der Vergleich mit den Angaben des Statistischen Bundesamts 2016 (Abb. 1) stellt zudem heraus, dass bislang durch das EUROCRINE®-Register lediglich ein geringer Anteil der in Deutschland durchgeführten Eingriffe an Nebennieren erfasst wurde. Weiterhin ließ die aktuelle Auswertung der Ergebnisse bislang keine Aussage über ein Langzeit-Follow-up (Tumorrezidiv, Gesamtüberleben) zu. Einträge über ausstehende Follow-up-Untersuchungen können jedoch prinzipiell zu späteren Zeitpunkten aus EUROCRINE® extrahiert werden, beispielsweise in einem zeitlichen Abstand von 5 oder 10 Jahren zu den erfolgten Operationen.

Die aktuelle Analyse ermöglicht einen Überblick über die aktuelle Versorgungsqualität der operativen Therapie von Nebennierenläsionen sowie über die tatsächliche Umsetzung der Leitlinien in den teilnehmenden Kliniken der deutschsprachigen EUROCRINE®-Länder. Durch das Aufzeigen bestehender Defizite kann eine Verbesserung der zukünftigen Dokumentation und Versorgung erreicht werden.

## Ausblick

Durch eine Zunahme der an EUROCRINE® teilnehmenden Kliniken kann europaweit eine genauere und umfassendere Auswertung unterschiedlichster Fragestellungen der endokrinen Chirurgie ermöglicht werden. Durch die Dokumentation durchgeführter Operationen in Zusammenschau mit Follow-up-Untersuchungen kann ein wichtiger Beitrag zu einem Erkenntnisgewinn hinsichtlich einer Optimierung operativer Verfahren geleistet werden. In naher Zukunft sind beispielsweise bereits weitere Analysen der Sensitivität und Spezifität der dokumentierten präoperativen diagnostischen Verfahren in Bezug auf die Feststellung der korrekten Detektion maligner Nebennierentumoren geplant.

Neben dem Anspruch der Verbesserung der operativen Therapie durch EUROCRINE®-basierte klinische Forschung kann die Datenbank durch die teilnehmenden Kliniken für ein individuelles Benchmarking genutzt werden, welches durch einen zeitlich unabhängig durchführbaren Datenexport in Microsoft Excel oder Microsoft PowerBI ermöglicht wird [21].

## Fazit für die Praxis

- Der Hauptanteil der Nebennierenoperationen im deutschsprachigen EUROCRINE®-Gebiet erfolgte in den Jahren 2015 bis 2019 bei der Diagnose „Nebennierenrindenadenom“.
- Bei Nebennierenmetastasen anderer Tumorentitäten wurden im Rahmen onkologischer Konzepte zahlreiche Adrenalektomien durchgeführt (dritthäufigste Diagnose des Kollektivs).
- Insgesamt 28 % der Nebennierenrindenkarzinome wurden einer minimal-invasiven Resektion zugeführt, mit einer Konversionsrate von 20 %.
- Die primäre Resektionsstrategie bei Nebenniereneingriffen stellte die unilaterale Adrenalektomie dar, welche in 83 % der Fälle minimal-invasiv (laparoskopisch > retroperitoneoskopisch) erfolgte.
- Robotisch assistierte Verfahren spielen im untersuchten Zeitraum von 2015 bis 2019 in Deutschland, Österreich und der Schweiz keine entscheidende Rolle.
